# DIFFERENCES AND SIMILARITIES OF ELDERLY PERSONS IN SWEDEN WITH A DIAGNOSIS OF PSYCHOSIS OR NON-PSYCHOSIS (SMI)

**DOI:** 10.1093/geroni/igac059.2867

**Published:** 2022-12-20

**Authors:** Per Bülow, Pia H Bülow, Deborah Finkel

**Affiliations:** Jönköping University, School of Health and Welfare, Jönköping, Jonkopings Lan, Sweden; School of Health and Welfare, Jönköping, Jonkopings Lan, Sweden; Indiana University Southeast, New Albany, Indiana, United States

## Abstract

Psychiatric care in Sweden is jointly organized by psychiatric practice and municipal social services. To determine who is entitled to support from the municipalities, the concept of “psychiatric disability” was created in connection with psychiatric reform in 1995. Psychiatric disability is a poorly identified concept and in Sweden, a person has severe mental illness (SMI) if they have difficulties in carrying out activities in crucial areas of life, these difficulties are caused by a mental disorder, and they are prolonged. Internationally, SMI is often synonymous with psychosis, but in Sweden other severe psychiatric conditions are included, but not dementia. Both practically and ethically, the unclear definition of SMI is a problem because it determines whether a person is granted interventions and what forms the interventions take. We investigated similarities and differences in people defined as SMI, divided into two groups, psychosis (Nf222) and non-psychosis (Nf253). Adults with SMI aged 65 or over (in 2016) have been assessed using data from four surveys carried out between 1996 and 2011, as well data available from national registers. People with psychosis had worse functional levels on the Global Assessment of Functioning and more unmet needs, according to Camberwell Assessment of Needs. However, differences between psychosis and non-psychosis groups varied across measures (e.g., education, income, living situation) and results differed depending on age at onset, year of first admission to a mental hospital, and length of institutionalization. These variables had a greater impact on the similarities and differences between measures than the diagnosis itself.

